# Revised Approach to the Role of Fatigue in Anterior Cruciate Ligament Injury Prevention: A Systematic Review with Meta-Analyses

**DOI:** 10.1007/s40279-019-01052-6

**Published:** 2019-01-18

**Authors:** Anne Benjaminse, Kate E. Webster, Alexander Kimp, Michelle Meijer, Alli Gokeler

**Affiliations:** 1 Center for Human Movement Science, University Medical Center Groningen, University of Groningen, Antonius Deusinglaan 1, 9713 AV Groningen, The Netherlands; 2 School of Sport Studies, Hanze University Groningen, Groningen, The Netherlands; 3 School of Allied Health, College of Science, Health and Engineering, La Trobe University, Bundoora, Melbourne, VIC Australia; 4 Midwifery Academy Amsterdam Groningen (AVAG), Groningen, The Netherlands; 5 Exercise Science & Neuroscience Unit, Department Exercise & Health, Faculty of Science, University of Paderborn, Paderborn, Germany; 6 Luxembourg Institute of Research for Orthopedics, Medicine and Science in Sports, Luxembourg City, Luxembourg

## Abstract

**Background:**

Causes of anterior cruciate ligament (ACL) injuries are multifactorial. Anterior cruciate ligament injury prevention should thus be approached from a multifactorial perspective as well. Training to resist fatigue is an underestimated aspect of prevention programs given that the presence of fatigue may play a crucial role in sustaining an ACL injury.

**Objectives:**

The primary objective of this literature review was to summarize research findings relating to the kinematic and kinetic effects of fatigue on single-leg landing tasks through a systematic review and meta-analysis. Other objectives were to critically appraise current approaches to examine the effects of fatigue together with elucidating and proposing an optimized approach for measuring the role of fatigue in ACL injury prevention.

**Methods:**

A systematic literature search was conducted in the databases PubMed (1978–November 2017), CINAHL (1992–November 2017), and EMBASE (1973–November 2017). The inclusion criteria were: (1) full text, (2) published in English, German, or Dutch, (3) healthy subjects, (4) average age ≥ 18 years, (5) single-leg jump landing task, (6) evaluation of the kinematics and/or kinetics of the lower extremities before and after a fatigue protocol, and (7) presentation of numerical kinematic and/or kinetic data. Participants included healthy subjects who underwent a fatigue protocol and in whom the effects of pre- and post-fatigue on three-dimensional lower extremity kinematic and kinetics were compared. Methods of data collection, patient selection, blinding, prevention of verification bias, and study design were independently assessed.

**Results:**

Twenty studies were included, in which four types of single-leg tasks were examined: the single-leg drop vertical jump, the single-leg drop landing, the single-leg hop for distance, and sidestep cutting. Fatigue seemed to mostly affect initial contact (decreased angles post-fatigue) and peak (increased angles post-fatigue) hip and knee flexion. Sagittal plane variables at initial contact were mostly affected under the single-leg hop for distance and sidestep cutting conditions whilst peak angles were affected during the single-leg drop jump.

**Conclusions:**

Training to resist fatigue is an underestimated aspect of prevention programs given that the presence of fatigue may play a crucial role in sustaining an ACL injury. Considering the small number of variables affected after fatigue, the question arises whether the same fatigue pathways are affected by the fatigue protocols used in the included laboratory studies as are experienced on the sports field.

**Electronic supplementary material:**

The online version of this article (10.1007/s40279-019-01052-6) contains supplementary material, which is available to authorized users.

## Key Points

**Table:** 

Current fatigue protocols might over-simplify a complex system.
An optimized approach to the role of fatigue in anterior cruciate ligament injury prevention might be necessary in which workload, aerobic fitness and fatigue serve as interacting factors.
The combination of practising open skills where athletes have to respond to unanticipated events in a fatigued condition may have merit given the similarity to demands in a game.

## Introduction

Injuries significantly impair both individual and team performance. Prevention must therefore be a priority [1]. As anterior cruciate ligament (ACL) injuries continue to rise per 1000 athlete exposures [2], there is a need for a critical appraisal of current injury prevention programs. Current ACL injury prevention programs typically involve a combination of plyometrics, strength training, agility, and balance exercises [3–5]. The key to avoiding an injury is the ability of an athlete to create stable motor output, even under sport-specific fatigued conditions in a complex athletic environment [6, 7], where all segments of the body act in synergy [8]. The pathway to fatigue runs parallel to the pathway to injury [8]. Both processes lead to a decrease of synergy of body segments during movement owing to, for example, coordinative changes, a reduction in degrees of freedom, or loss of efficiency [8]. However, training to resist fatigue is typically not included in injury prevention protocols, even though the presence of fatigue may play a role in sustaining an ACL injury [9].

**Fig. 1:**
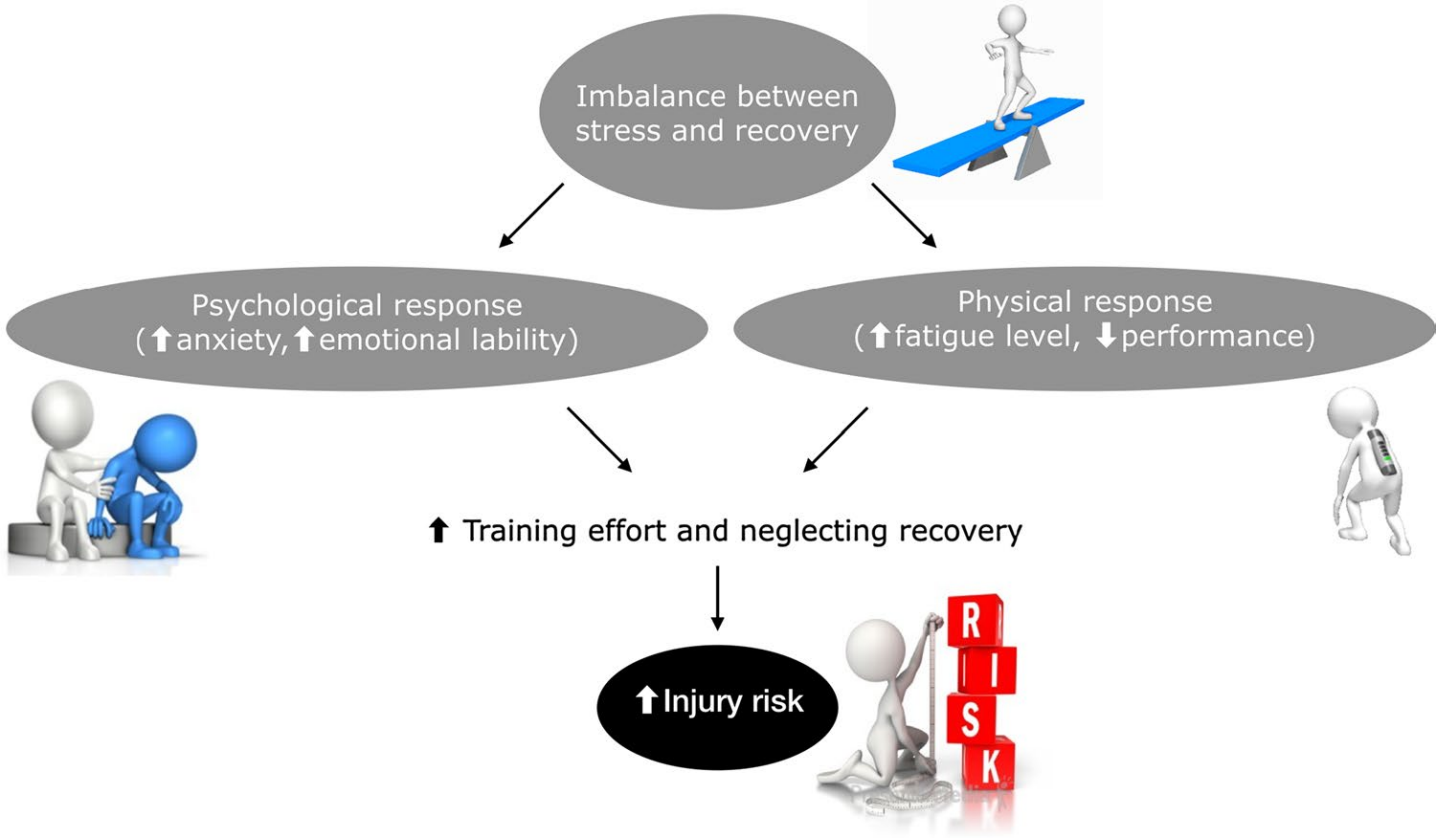
Illustration of mechanisms of fatigue that can increase injury risk

The currently most used measure of fatigue is incremental fatigue related to playing time [10]. However, injury surveillance data have not shown a consistent relationship between fatigue as a result of playing time and injury [11]. This approach may be too simple and an important perspective to include in an injury prevention model is the fact that an imbalance between stress and recovery can generate several physical (e.g., increased fatigue level, decreased performance) and psychological (e.g., increased anxiety, emotional lability) responses [9] (Fig. 1). Athletes can respond in two ways to an imbalance between stress and recovery. Either they adjust their activities (i.e., increasing recovery and decreasing training load) and return to a balance between stress and recovery, or they ignore the physical and psychological reactions (i.e., increasing training effort and neglecting recovery), which is generally associated with adverse outcomes, such as an increased likelihood of becoming injured and an increased risk for both overtraining syndrome and chronic fatigue [9, 12]. Additionally, for instance, increases in pre-surgery stress have been shown to negatively impact on both rehabilitation compliance and knee symptoms [13, 14].

To date, laboratory studies have shown conflicting results pertaining to the effect of fatigue on lower limb biomechanics during athletic tasks [15, 16]. However, these laboratory studies do not reflect the complexity of physical and psychological fatigue that occurs during an actual game [17], which may be a reason for these conflicting results.

This complexity can be demonstrated in three examples. First, fatigue can occur early in a game when an athlete has not had enough sleep the night before the game day or has heightened levels of stress/daily hassles. In this situation, suboptimal recovery makes the athlete perceive a higher internal workload and feel more fatigued. This increased fatigue might make the athlete more vulnerable to injury [9, 18, 19]. Second, athletes can experience fatigue after a sudden 1-min spike in acute workload during the game [17, 20]. Third, an athlete can experience neuromuscular fatigue as a result of playing time (i.e., workload) [21–23] and thus be more vulnerable as the game progresses. These three examples display the complexity of factors interacting with each other. Training to resist fatigue is an underestimated aspect of prevention programs given that the presence of fatigue may play a crucial role in sustaining an ACL injury.

Our understanding of the concept of fatigue in relation to injury prevention may thus need to be revised in relation to the ACL injury risk profile. With a better understanding, we may be able to increase the external validity of testing the effects of fatigue and eventually assist in more effective implementation of injury prevention programs for ball team sport athletes.

The primary objective of this systematic review and meta-analysis was to summarize research findings relating to the kinematic and kinetic effects of fatigue on single-leg landing tasks. Other objectives were to critically appraise current approaches to examining the effects of fatigue together with elucidating and proposing an optimized approach for measuring the role of fatigue in ACL injury prevention.

The article is divided into two sections. First, we present the systematic review and meta-analysis (Sects. 2, 3, and 4.1–4.3). Second, we critically discuss the current methods of measuring the role of fatigue in ACL injury prevention and present a revised approach to injury prevention (Sects. 4.4–4.8).

### Definitions

#### Psychological and Physical Fatigue

Fatigue can be defined as the decrease in the pre-match/baseline psychological and physiological function of the athlete [24]. The factors that cause someone to move in a particular way, which may increase their risk of injury, constitute a complex relationship between psychological and physical factors.

For example, when an athlete has to cope with psychological stress (i.e., external psychological load), this can affect perceptual abilities (i.e., experienced internal load), e.g., central and peripheral vision and reaction time [25, 26]. When alertness is decreased, attention and decision making will be reduced because of psychological fatigue. Athletes may be unable to respond in a timely fashion to the abundant somatosensory information and the biomechanical demands of a rapidly changing physical environment [6], such that movement patterns may become detrimental [27].

However, external physical load [28] can be perceived differently by each individual athlete (i.e., experienced internal load) [17]. For example, a biomechanical load with accelerations and decelerations when landing from a jump or sidestep cutting needs to be countered by a reverse optimal internal (joint) load. Absorption of external load has been shown to be associated with clinically relevant biomechanical deficits when individuals are fatigued [23]. Thus, a given external workload is a poor predictor of fatigue because individuals vary widely in their internal response [17].

#### Physical: Central and Peripheral Fatigue

It is common to distinguish between central fatigue and peripheral fatigue [29, 30]. Central fatigue refers to an exercise-induced reduction in the level of voluntary muscle activation [29, 30] (i.e., reduced central drive, autonomic nervous system alterations, and neuromuscular fatigue) as a result of impairments proximal to the neuromuscular junction [29, 30]. Peripheral fatigue refers to exercise-induced processes leading to a reduction in the force-generating capacity of the muscle (i.e., metabolic and mechanical damage and neuromuscular fatigue) occurring at or distal to the level of the neuromuscular junction [29, 30].

## Methods

### Literature Search

A systematic literature search was conducted in the databases PubMed (1978–November 2017), CINAHL (1992–November 2017), and EMBASE (1973–November 2017) (Table 1). A combination of the following search terms was used: (1) fatigue, (2) knee joint, lower limb, leg, knee, hip, ankle, (3) kinetics, kinematics, biomechanics, and (4) land*, jump*, side*, step*, single, cut*, task*, task performance. Within groups, the search terms were combined with the OR operator; between groups, search terms were connected with the AND operator. The results of the three searches were combined and duplicates were removed. These electronic searches were supplemented by manual searches and cross-checking the reference lists and citations of relevant published studies (i.e., checking on search terms, inclusion criteria, activities and/or population in the title and abstract).

**Table 1:** Search strings and terms per database

PubMed (1978–November 2017)	CINAHL (1992–November 2017)	EMBASE (1973–November 2017)
“Fatigue” AND (“Knee joint” OR “Knee” OR “Lower limb” OR “Leg” OR “Hip” OR “Ankle”) AND (“Kinetics” OR “Kinematics” OR “Biomechanics”) AND (“Land*” OR “Jump*” OR “Single” OR “Task*” OR “Task performance”)	“Fatigue” AND (“Knee joint” OR “Knee” OR “Lower limb” OR “Leg” OR “Hip” OR “Ankle”) AND (“Kinetics” OR “Kinematics” OR “Biomechanics”) AND (“Land*” OR “Jump*” OR “Single” OR “Task*” OR “Task performance”)	‘Fatigue’ AND (‘Knee joint’ OR ‘Knee’ OR ‘Lower limb’ OR ‘Leg’ OR ‘Hip’ OR ‘Ankle’) AND (‘Kinetics’ OR ‘Kinematics’ OR ‘Biomechanics’) AND (Land* OR Jump* OR ‘Single’ OR ‘Task’ OR ‘Task performance’)

After an initial review by M.M., all irrelevant papers were excluded. Full texts were independently analyzed by two authors (A.B. and M.M.) for final inclusion, based on predefined inclusion and exclusion criteria. Any discrepancy was resolved by a consensus meeting between the two reviewers. If this failed to resolve the issue, the opinion of a third person was sought (K.W.). The inclusion criteria were: (1) full text, (2) published in English, German, or Dutch, (3) healthy subjects, (4) average age ≥ 18 years, (5) single-leg landing task, (6) evaluation of the kinematics and/or kinetics of the lower extremities before and after a fatigue protocol, and (7) presentation of numerical kinematic and/or kinetic data. Participants included healthy subjects who underwent a fatigue protocol and in whom the effects of pre- and post-fatigue on three-dimensional (3D) lower extremity kinematics and kinetics were compared.

### Data Extraction and Analysis

The following data were extracted and summarized from each included article: characteristics of the subjects, landing task, fatigue protocol, study design and outcome measures, results, and key findings. The measures of interest were pre- and post-fatigue 3D joint angles and moments of the hip, knee, and ankle at landing. The data were independently extracted by three reviewers (A.B., M.M., A.K.) [Tables S1–S4 of the Electronic Supplementary Material (ESM)]. Again, any discrepancy was resolved by a consensus meeting between the two reviewers. If this failed to resolve the issue, the opinion of a fourth person was sought (K.W.). Effect size (ES) meta-analyses using StatsDirect Ltd, Cambridge, UK were conducted for each primary variable for which there were a minimum of three samples. A minimum of three samples was chosen because of the large number of possible 3D biomechanical outcomes and to better identify consistency of findings. For all analyses, the DerSimonian and Laird random-effects model was used owing to the heterogeneity of the study samples. All analyses are expressed using 95% confidence intervals (95% CIs) and Cohen’s ES statistic (Cohen’s *d*) where *d* = 0.2–0.5, *d* = 0.5–0.8, and *d* ≥ 0.8 represent small, moderate, and large effects, respectively [31].

### Risk of Bias in Individual Studies

To evaluate the validity of the studies and the applicability of the results (items b–f), the methodological quality of all included studies was assessed with the modified scoring list based on the Cochrane Group on Screening and Diagnostic Test Methodology [32]. The Downs and Black revised checklist was used for measuring study quality (items g–q) [33]. Methods of data collection, subject selection, blinding, prevention of verification bias, and study design were independently assessed. The reviewers agreed on the answers to all these questions.

## Results

### Methodological Quality and Study Characteristics

**Fig. 2:**
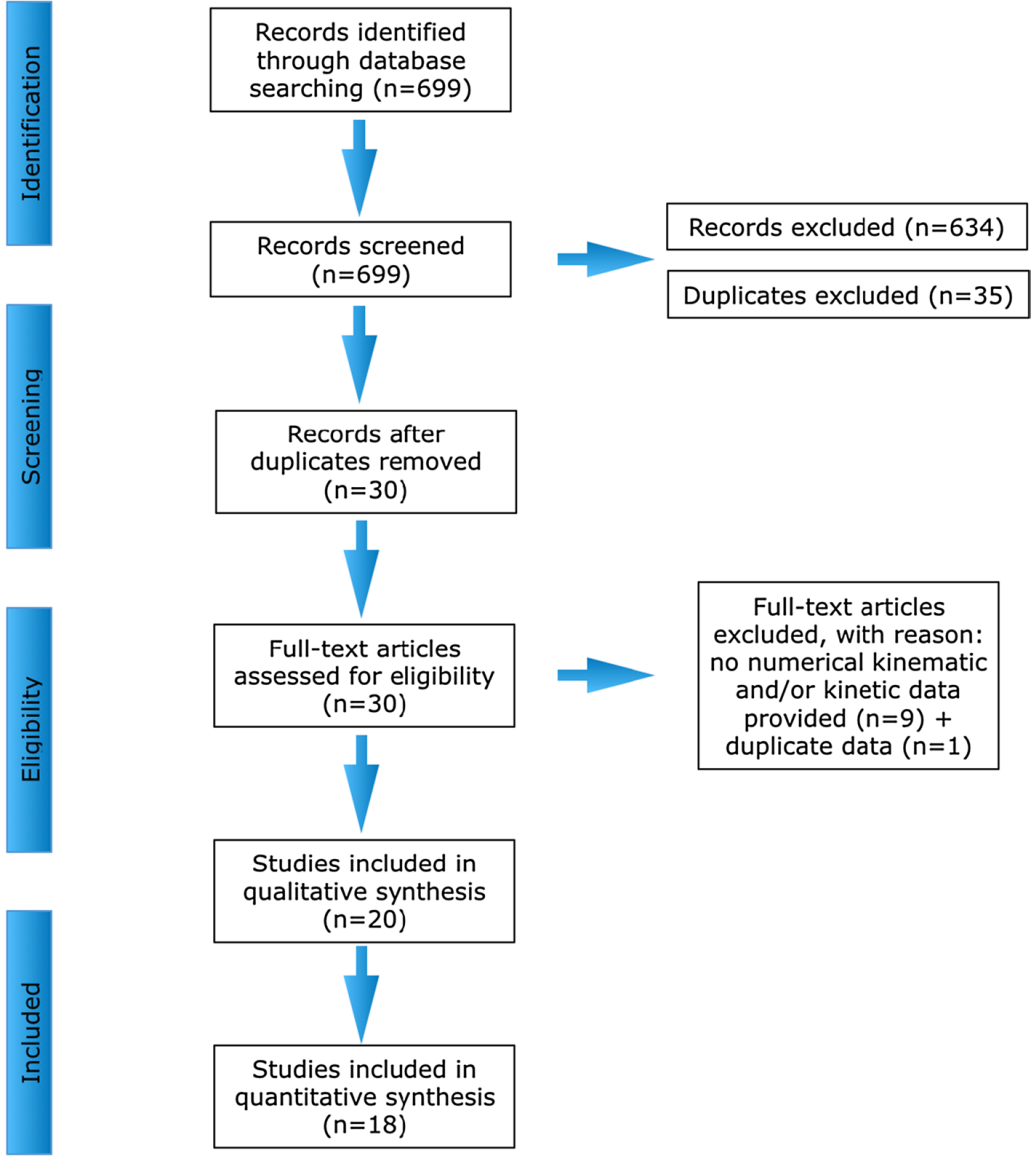
Flow chart of study selection

The searches in PubMed, EMBASE, and CINAHL revealed 177, 406, and 116 studies, respectively. Of these studies, 634 studies were excluded (not relevant as they did not cover the main topic, activities and/or population), 35 duplicates were removed. Nine studies lacking kinematic and/or kinetic data were excluded. One study was excluded [54] because it contained duplicate data from another study [53]. Twenty studies were included for review (Fig. 2), of which two studies were excluded from the meta-analyses, as not enough data samples were available from these studies [34, 35]. The four types of single-leg tasks in this review were: (1) single-leg drop vertical jump (SLDVJ, *n* = 5 studies) [36–40], (2) single-leg drop landing (SLDL, *n* = 8 studies) [41–48], (3) single-leg hop for distance (SLHD, *n* = 3 studies) [34, 49, 50], and (4) sidestep cutting (SSC, *n* = 4 studies) [35, 51–53].

A detailed description of the methodological quality and characteristics of the studies included in this review is presented in Table S5 of the ESM and Table 2. Fifteen studies conducted central fatigue protocols [35–43, 45, 48, 49, 51, 52, 54] and five studies conducted peripheral fatigue protocols [34, 44, 46, 47, 50]. Six studies included both female and male subjects [37, 41, 43, 44, 48, 50], nine studies included only female subjects [35, 38, 40, 45–47, 51, 52, 54], and five studies included only male subjects [34, 36, 39, 42, 49]. The average age of included subjects was 24.89 ± 4.26 years and 20.68 ± 1.35 years for male and female subjects, respectively. The number of participants per study included in the review ranged from 8 (male subjects) [34] to 40 (20 female subjects and 20 male subjects) [48]. The overall quality score ranged from 12 to 17 (maximum 21). Most studies were level 4 studies, but two studies were level 1 [38, 40]. This was mainly because they included a control group and assigned subjects to the groups randomly. Only two studies took confounders into account [34, 50]. Nine out of the 20 studies reported power calculations [39, 43, 45–47, 50–52, 54], calculated as 0.8 and 0.9.

**Table 2:** Study characteristics

Study, year	Sex, age (years)	Sport	Level	Task (A/UA)	Instruction given	Fatigue protocol (C/P)	Study design	Measure of fatigue	Outcome measures
Single-leg drop vertical jump									
Coventry et al., 2006 [36]	10 M (23.8 ± 2.4)	Physically active	Recreational (i.e., at least 30 min, most days of the week)	Max single-leg CMJ after landing (A)	Instructed to perform a series of jumps on a force plate	Cycles of landing, CMJ, 5 single-leg squats to 90° knee flexion (C)	Repeated cycles until fatigued	Until participants ‘could not stick the next landing’	vGRF peak and loading rate Hip, knee, ankle FLEX/EXT angle IC, peak. and RoM Hip, knee, ankle peak angular velocity, peak FLEX/EXT moment, and peak negative power
Benjaminse et al., 2008 [37]	15 M (22.7 ± 1.6)/15 F (22.1 ± 1.7)	Healthy and physically active (aerobic exercise)	Recreational (i.e., at least three times a week for at least 30 min/d)	Single-leg standing stop jump immediately followed by a max effort vertical jump (A)	Instructed on proper start position, single-leg landing on the force plate and maximum effort vertical jump	Treadmill: 3-min warm-up 2 mph, 3-min incr. speed (5–8 mph), incr. grade with 2.5% every 2 min (C)	5 jumps, fatigue protocol, 5 jumps	Until ‘subject could not run anymore at maximum effort’	Knee FLEX/EXT angle IC and peak Hip IR/ER angle IC and peak Knee ABD/ADD angle IC and peak
Tamura et al., 2016 [38]	34 F (20.7 ± 1.8)	NA	NA	Single-leg drop vertical jump (A)	Testing sequence was shown by an assistant researcher	Bike ergometer 100W 5 min (C)	5 single-leg drop vertical jumps, fatigue protocol, 5 single-leg drop vertical jumps	Until exceeding 17 on Borg scale	Knee FLEX/EXT angle peak
Lessi et al., 2017 [39]	20 M (22.8 ± 2.9)	Anyone participating in aerobic or athletic activity	Recreational (i.e., at least three times a week)	Single-leg drop vertical jump (A)	Instructed to hold arms across their chest, step off box without jumping up, stepping down or losing balance, and land with the dominant limb + no verbal or visual clues were given for the landing techniques at any time	10 bilateral squats (90 knee flexion), 2 bilateral max effort vertical jumps and 20 steps (31-cm-high stair) (C)	3 single-leg drop vertical jumps, fatigue protocol, 3 single-leg drop vertical jumps	Until hop distance was reduced at least by 20%	Hip, knee FLEX/EXT, ABD/ADD angle IC, and peak
Tamura et al., 2017 [40]	34 F (20.7 ± 1.8)	NA	NA	Single-leg drop vertical jump (A)	Testing sequence was shown by an assistant researcher	Bike ergometer 100W 5 min (C)	5 single-leg drop vertical jumps, fatigue protocol, 5 single-leg drop vertical jumps	Until exceeding 17 on Borg scale	Hip, knee, ankle FLEX/EXT angle peak, IC, 40 ms after IC, at vGRF
Single-leg drop landing									
Madigan et al., 2003 [42]	12 M (27.9 ± 5.4)	Subjects who were physically active	Recreational	Single-leg drop landing (A)	Instructed to land on a visual target placed 33 cm from the front edge of the elevated platform using a toe-to-heel strategy	Single-leg squats (C)	Single-leg drop landing, 2 single-leg squats, single-leg drop landing, until fatigued	Until subjects felt their right knee would collapse upon the next landing	vGRF peak, impulse and max loading rate Hip, knee, ankle FLEX/EXT angle IC, and peak Hip, knee, and ankle angular impulse
Kernozek et al., 2008 [43]	16 M (23.8 ± 0.4)/14 F (23.0 ± 0.9)	1 or more sports, such as tennis, basketball, volleyball, and soccer	Recreational (i.e., at least two times a week)	Single-leg 50-cm drop landing (A)	Instructed to land as normally and as comfortably as possible without falling, losing balance, stepping off the plate, or touching the ground with either hand	Sets squats (60% of 1 RM) (C)	6 single-leg drop landings, fatigue protocol, 6 single-leg drop landings	Until failure occurred (when they had completed 4 or more sets and could no longer lift the weight)	Hip, knee, ankle FLEX/EXT angle, and moment peak hip, knee, ankle ABD/ADD angle and moment peak vGRF
Kellis and Kouvelioti, 2009 [44]	10 M (24.3 ± 1.25)/10 F (23.5 ± 1.43)	Physical education students	NA	Single-leg drop landing from 30-cm height (A)	Instructed to perform several single-leg landings from a 30-cm drop height on the force plate with hand on the hips + no verbal or visual reinforcement was provided during the tests	2 sets of consecutive concentric efforts of the knee extensors or flexors on a dynamometer (P)	10-min cycle warm-up, jumps, first part fatigue protocol, jumps, second part fatigue protocol, jumps	Until the subjects could no longer produce 30% of the maximum moment	Hip, knee FLEX/EXT angle IC, and peak vGRF
McLean and Samorezov, 2009 [45]	20 F (19.2 ± 1.7)	Volleyball, soccer, and basketball	NCAA Division 1	Single-leg drop landing (UA)	Instructed to perform one of three randomly ordered jump landings, with the jump initiated from a stationary starting position located 2 m behind the force plates	Set of 3 single-leg squats immediately followed by a randomized landing trial (C)	Performing 6 jumps (un)anticipated, fatigue protocol, performing 6 jumps (un)anticipated	Until subjects could no longer complete three sequential squats unassisted	Hip, knee FLEX/EXT angle IC, and PS Hip IR/ER angle IC and PS Knee ABD/ADD and IR/ER angle PS Hip, knee FLEX/EXT and IR/ER moment PS Knee ABD/ADD moment PS
Brazen et al., [41] 2010	12 M (21.3 ± 2.8)/12 F (19.5 ± 1.7)	Collegiate or club athletics or actively participated in intramural university sports	NA	Single-leg drop landing (A)	Instructed on how to perform a single-leg drop landing onto a force plate in a natural position	Agility drills, side-to-side bounds, minitrampoline jumps, minihurdle hops, vertical jumps (C)	3 jumps, 4-min warm-up on a cycle ergometer and stretching exercises, fatigue protocol, 3 jumps	6 rounds of the fatigue protocol or when they felt unable or if any visible signs of exhaustion were shown including but not limited to shortness of breath, chest pain, or confusion	Time to stabilization Knee, ankle FLEX/EXT angle IC Knee ABD/ADD angle IC vGRF
Patrek et al., 2011 [46]	20 F (21.0 ± 1.3)	Participating in aerobic or athletic activity	At least three times a week for at least 30 min/d/7 athletes were NCAA Division III track-and-field	Single-leg drop landing (A)	Instructed to land as comfortably and normally as possible without falling over, stepping off the force platform, or touching the ground with either their hands or non-dominant leg	Hip abductor fatigue protocol (P)	Baseline strength test, 5 jumps, fatigue protocol, 5 jumps	Borg RPE of 19 or greater (on a 6–20 scale) and when failed to touch the bar on 2 consecutive repetitions at the proper tempo	Hip, knee FLEX/EXT angle, ABD/ADD angle IC and 60ms after IC Hip, knee FLEX/EXT and ABD/ADD moment IC and 60ms after IC vGRF
Thomas et al., 2011 [47]	16 F (18–22)	Recreationally active volunteers	Recreational	Single-leg drop landing (A)	Instructed to jump forward off both legs over a box and land with the dominant limb centered on force platform	Hip rotators fatigue protocol, ipsilateral triceps surae fatigue protocol (P)	3 jumps, fatigue protocol, 3 jumps	When the first five maximum voluntary concentric contractions of any given set were performed 80% below the baseline peak torque measure	Hip, knee, ankle FLEX/EXT angle IC, and PS Hip, knee ABD/ADD and IR/ER angle PS and IC Ankle INV/EV angle IC and PS Hip, knee, ankle FLEX/EXT, ABD/ADD, IR/ER moment PS Ankle moment INV/EV PS
Liederbach et al., 2014 [48]	20 M (22.0 ± 2.0)/20 F (20.0 ± 2.0)	40 dancers and 40 team sport athletes	NA	Single-leg drop landing (A)	NA	50 step-ups on 30-cm box and 15 max effort single-leg vertical jumps (C)	Single-leg drop landing, fatigue protocol, single-leg drop landing	Until a 10% decrement in maximum vertical jump height	Hip, knee FLEX/EXT, ABD/ADD angle IC and peak Knee ABD/ADD moment IC and peak Knee FLEX/EXT moment peak Hip IR/ER ankle IC and peak Hip IR/ER moment peak
Single-leg hop for distance									
Augustsson et al., 2006 [34]	8 M (31.0 ± 6.0)	Generally physically active	NA	Single-leg hop for distance (A)	Instructed to hop forward as far as possible and to land on the same leg + allowed to swing arms freely	Consecutive unilateral knee extension with a load of 50% and 80% of 1 RM (P)	Single-leg hops, fatigue protocol, jumps, 3-min recovery, jumps	Until failure occured	Hip, knee, ankle FLEX/EXT angle peak Hip, knee, ankle FLEX/EXT moment peak Hip, knee, ankle generated power GRFx GRFy GRFz GRFyz
Orishimo and Kremenic, 2006 [49]	13 (33.9 ± 7.2)	NA	NA	Single-leg hop for distance (A)	NA	At least two sets of 50 step-ups (C)	Single-leg hops for distance, fatigue protocol, single-leg hop for distance, until 80% of their pre-fatigue max distance	80% maximum hop distance	Hip, knee, ankle FLEX/EXT range Hip, knee, ankle FLEX/EXT moment peak Hip, knee, ankle peak power Peak vGRF
Thomas et al., 2010 [50]	13 M (20.31 ± 0.85)/12 F (20.33 ± 1.33)	Recreational volunteers	Recreational	Single-leg hop for distance (A)	Instructed to jump forward off and land on their dominant leg on the force platform	Alternating QH MVCC (P)	3 hops, fatigue protocol, 3 hops	Until the torque measured in both muscle groups dropped below 50%	Hip, knee FLEX/EXT angle and moment IC and peak vGRF Hip, knee ABD/ADD angle and moment IC and peak vGRF Hip, knee IR/ER angle and moment IC and peak vGRF
Sidestep cutting									
Sanna and O’Connor, 2008 [51]	12 F (20.1 ± 1.2)	Soccer	NCAA Division 1	Sidestep cutting (A)	Instructed to cut at 45° with the stance foot landing on the force plate	Intermittent shuttle run test (60 min) (C)	Preliminary: 20-m progressive shuttle run test, practice CMJ and SCM, 5 + 3 jumps, fatigue protocol, 5 + 3 jumps	60 min for each subject	Hip, knee, ankle FLEX/EXT, ABD/ADD and IR/ER angle at peak and RoM Hip, knee, ankle FLEX/EXT, ABD/ADD and IR/ER moment at peak
Lucci et al., 2011 [52]	15 F (19.2 ± 0.8)	Soccer	NCAA Division 1	Unanticipated sidestep cutting (UA)	Receiving visual cue of two soccer scenarios, the ball cutting to one side and the ball stopping	FAST-FP and SLO-FP (C)	5 jumps, fatigue protocol, 5 jumps	SLO-FP: When the participants felt they were maximally fatigued and could no longer continue running FAST-FP: The subjects had to perform a total of four sets of the fatiguing protocol with no rest in between	Hip FLEX/EXT and IR/ER angle at IC, PS, PVGRF and PPGRF Knee FLEX/EXT and IR/ER at IC, PS, PVGRF, PPGRF and PKF Knee ABD/ADD angle IC PVGRF PPGRF PKF Knee FLEX/EXT and ABD/ADD moment at IC and PS Hip ABD/ADD moment at IC vGRF
Cortes et al., 2013 [53]	18 F (19.2 ± 0.9)	Soccer	NCAA Division 1	Unanticipated sidestep cutting (UA)	NA	FAST-FP (C)	3 CMJ at 90% of max vertical jump, step ups, step downs on a 30-cm box for 20 s, 3 squats to 90° of knee flexion, + proagility shuttle run (5-10-5 agility run)	FAST-FP: The subjects had to perform a total of four sets of the fatiguing protocol with no rest in between	Knee and hip FLEX/EXT angle at IC and PS Knee and hip ABD/ADD angle at IC Knee and hip ABD/ADD moment at IC and PS Hip ABD/ADD moment at IC and PS
Collins et al., 2016 [35]	13 F (21.6 ± 2.2)	Soccer	NCAA Division 1	Unanticipated sidestep cutting (UA)	Instructed to cut along a 1-m-wide path	Intermittent shuttle run test (60 min) (C)	Calibration: 5 CMJ, 15 unanticipated sidestep cutting, 15 preplanned sidestep cutting, fatigue protocol, calibration: 5 CMJ, 15 unanticipated sidestep cutting, 15 preplanned sidestep cutting	Lasts 60 min for each subject	Knee ABD/ADD angle peak Knee FLEX/EXT angle peak Knee IR/ER angle peak Knee FLEX/EXT and ABD/ADD and IR/ER moment peak

*A* anticipated, *ABD/ADD* abduction/adduction, *CMJ* counter movement jump, *F* female, *FAST-FP* functional agility short-term fatigue protocol, *FLEX/EXT* flexion/extension, *C* central, *GRFx* ground reaction force horizontal, *GFRy* ground reaction force medio-lateral, *GRFz* ground reaction force vertical, *GRFyz* ground reaction force resultant vector of horizontal and vertical forces, *IC* initial contact, *INV/EV* inversion/eversion, incr increase, *IR/ER* internal rotation/external rotation, *P* peripheral, *M* male, max maximal, *MVCC* maximum voluntary concentric contractions, *NA* not applicable, *NCAA* National Collegiate Athletic Association, *PKF* peak knee flexion, *PPGRF* peak posterior ground reaction force, *PS* peak stance, *PvGRF* peak vertical ground reaction force, *QH* quadriceps and hamstrings, *RM* repetition maximum, *RoM* range of motion, *RPE* rate of perceived exertion, *SCM* sidestep cutting maneuvers, *SLO-FP* slow linear oxidative fatigue protocol, *UA* unanticipated, *VGRF* vertical ground reaction force

### Pooled Analysis

The pooled effects of fatigue for the sagittal plane are presented in Tables 3 and 4 and in Figs. S1–S4 of the ESM for knee flexion angle at initial contact (IC), peak knee flexion angle, hip flexion angle at IC, and peak hip flexion angle, respectively. Knee flexion angle at IC was significantly smaller post-fatigue during the SLHD (*p* = 0.001, ES = 0.84, 95% CI 0.34–1.34) and SSC (*p* = 0.0101, ES = 0.48, 95% CI 0.11–0.84). Hip flexion angle at IC significantly decreased post-fatigue during SSC (*p* = 0.016, ES = 0.45, 95% CI 0.08–0.81). Peak knee (*p* = 0.0005, ES = − 1.27, 95% CI − 1.98 to − 0.56) and hip (*p* = 0.0023, ES = − 0.48, 95% CI − 0.80 to − 0.17) flexion angles were significantly greater post-fatigue during the SLDL.

**Table 3:** Pooled effects of fatigue on initial contact (IC) and peak knee and hip flexion angles

Task	Effect of fatigue
Knee flexion IC (°)	
Single-leg drop vertical jump	NS
Single-leg drop landing	NS
Single-leg hop for distance	Decrease post-fatigue
Sidestep cutting	Decrease post-fatigue
Knee flexion peak (°)	
Single-leg drop vertical jump	NS
Single-leg drop landing	Increase post-fatigue
Single-leg hop for distance	–
Sidestep cutting	–
Hip flexion IC (°)	
Single-leg drop vertical jump	NS
Single-leg drop landing	NS
Single-leg hop for distance	NS
Sidestep cutting	Decrease post-fatigue
Hip flexion peak (°)	
Single-leg drop vertical jump	NS
Single-leg drop landing	Increase post-fatigue
Single-leg hop for distance	–
Sidestep cutting	–

*NS* not significant

**Table 4:** Pooled effects of fatigue on peak knee and hip frontal plane angles and moments

Task	Effect of fatigue
Knee abduction peak (°)	
Single-leg drop vertical jump	NS
Single-leg drop landing	NS
Single-leg hop for distance	–
Sidestep cutting	–
Hip abduction peak (°)	
Single-leg drop vertical jump	NS
Single-leg drop landing	NS
Single-leg hop for distance	–
Sidestep cutting	–
Knee abduction peak (Nm/kg)	
Single-leg drop vertical jump	NS
Single-leg drop landing	NS
Single-leg hop for distance	–
Sidestep cutting	–
Hip abduction peak (Nm/kg)	
Single-leg drop vertical jump	NS
Single-leg drop landing	–
Single-leg hop for distance	–
Sidestep cutting	–

*NS* not significant

The pooled effects of fatigue for the frontal plane for peak knee abduction/adduction angle and peak hip abduction/adduction angle are presented in Tables 3 and 4 and Figs. S5–S7 of the ESM. No significant overall effects were found.

The peak knee abduction moment decreased post-fatigue for the SLDL; however, this was non-significant (*p* = 0.2369, ES = 0.28, 95% CI − 0.18 to 0.74) [Fig. S7 of the ESM]. No further significant differences (e.g., at the ankle joint, kinetic differences, or in the frontal plane) because of fatigue were observed.

## Discussion

### Summary of Research Findings

The main finding was that fatigue had no significant impact on most of the kinetic and kinematic variables associated with the employed fatigue protocols included in this meta-analysis. This is consistent with other reviews [15, 16]. However, fatigue did induce a change in movement in the sagittal plane. Fatigue mostly affects IC (decreased angles post-fatigue) and peak (increased angles post-fatigue) hip and knee flexion. The stiffer landing strategy after fatigue at IC is similar to previous findings in that less knee flexion and greater vertical ground reaction force may place athletes at greater risk of ACL injury [55, 56]. It needs to be noted that the landing strategies of Leppänen et al. [56] have been observed during unfatigued double-leg drop vertical jump tasks and can thus not be directly compared. Which components of a 3D whole-body motion contribute mostly to joint load certainly depends on the task and manner in which someone is executing this task [56–60]. Fatigue of the quadriceps impairs motor coordination and makes it more difficult to eccentrically control deceleration of the knee [8, 61].

### Fatigue Protocols

Most fatigue protocols included vertical and sagittal movements, which could be a reason why significant overall effects were found only in the sagittal plane. Based on our meta-analyses, fatigue did not affect hip and knee abduction or adduction angles and moments. Although some individual studies found a significant effect of fatigue, none of the pooled overall effects for the frontal plane peak angles and moments (SLDVJ and SLDL) reached significance (Table 4) [37, 39, 43, 48]. However, a trend to a decreased peak knee abduction angle post-fatigue was observed for the SLDVJ (Fig. S5 of the ESM). For the SLDL, trends were seen for an increase in peak knee abduction angle (Fig. S5 of the ESM) and a decrease in peak knee abduction moment (Fig. S7 of the ESM) post-fatigue.

A wide variety of methods was used to collect kinematic and kinetic variables in the studies included in the review. Furthermore, the applied fatigue protocols and operational definitions of fatigue were very heterogeneous with no protocol or definition being the same across the studies. No clear trend for the effects of central vs. peripheral fatigue was found (Figs. S1–S7 of the ESM). Central fatigue protocols such as treadmill and bike ergometer, agility drills, squats, jumps and step-ups were used. Peripheral fatigue protocols contained mostly local hip or knee alternating flexion extension or hip abduction-adduction movements against resistance. Besides the different protocols and different subjective and objective measures of fatigue used in the studies, other factors such as individual physical fitness and coordination could have affected study results as well.

Of note is that most studies used preplanned tasks. However, research has shown that movement mechanics change unfavorably during unanticipated execution of a task compared with when the task is anticipated [35, 45, 62, 63]. Potentially, this more closely reflects aspects of a real game where the environment constantly changes and thus athletes must anticipate and adopt appropriate movement strategies. The integrative impact of fatigue and decision making may present a suboptimal combination for high-risk dynamic landing strategies [64]. That is, the demands of the sports environment allow athletes only milliseconds to perform the cognitive processing involved in movement selection (‘decision making’) [65, 66]. Not surprisingly, athletes with slower baseline cognitive processing speeds (e.g., longer reaction times) demonstrate mechanics that may result in greater ACL loading during execution of unplanned landing and cutting maneuvers [67–69]. Fatigue induced by intense exercise may result in decrements in cognitive processing (indicative of a ‘supraspinal’ effect) [70–76]. In addition, specific cognitive functions, such as concentration, deteriorate when experiencing higher stress levels, decreasing an individual’s ability to perform well in tasks that require high levels of attentional control (being ‘in the game’) [77]. Considering the important role that efficient cognitive processing appears to play in controlling movement in sports, potential fatigue-related transient decrements in cognitive functioning could compromise an athlete’s ability to maneuver within dynamic environments without injury.

It is also important to question whether a fatigue protocol until exhaustion [41] reflects sports-specific physiological loads [78]. For example, in soccer, landing after heading a ball or cutting to pass an opponent typically is not carried out by the player in a state of maximal exhaustion. Studies measuring rate of perceived exertion (RPE) using the Borg scale during or immediately after a soccer game in young, adolescent, male professional soccer players report RPE values between ‘hard’ and ‘very hard’, which indicates that players were not completely exhausted [23]. Others found that fatigue increased during a typical soccer game (from 2.2 during the first 10 min to 3.6 in the last 10 min on a 7-point scale) [79]. Only a minor decrease in fatigue was experienced during half-time, with attackers experiencing more fatigue than defenders [79]. Borotikar et al. [64] showed that biomechanical adaptations (i.e., increase in IC hip extension angle and peak knee abduction angle) are seen already at the 50% level of fatigue.

### Fatigue Effects on the Different Tasks

No significant overall effects of fatigue were found for the SLDVJ. After fatigue, greater overall peak knee (ES = − 1.27) and hip (ES = − 0.56) flexion angles were observed during the SLDL. It is worth mentioning that an increase in knee abduction angle during peak stance was found from an anticipated to an unanticipated SLDL task (− 3.4° ± 3.6° to − 7.2° ± 3.2°, respectively; *p* < 0.05, ES = − 1.20) [45]. This may indicate the relevance of adding sport-specific elements to testing and further shows the role of fatigue in decision making. During the SLHD, smaller knee flexion angles at IC were observed after fatigue, with a large ES (0.84). Last, during SSC, athletes showed a movement strategy with overall smaller hip (ES = 0.45) and knee (ES = 0.48) flexion angles at IC after fatigue. For both the SLHD and SSC, this stiffer landing technique may place the athlete at a greater risk for injury. Considering the ESs, it seems that the sagittal motion of the knee joint is most strongly affected, especially during the SLDL and SLHD. Again, this can be owing to the quadriceps having difficulty eccentrically controlling the required downward motion. To further clarify some of the potential differences, further research would be needed, including between task comparisons within cohorts.

### Summary

In conclusion, healthy athletes deal well with induced fatigue as observed in the included studies without observable detrimental biomechanical changes. Therefore, the construct validity of current fatigue protocols probably needs to be revised. Recently, it has been found that during the progression of a simulated soccer game, the overall RPE was not reflected in kinematic and kinetic changes during a countermovement jump and a single-leg drop jump [23]. This suggests that the protocol was predominantly centrally demanding and peripheral control was not reduced. Another explanation could be that similar pathways are affected, but the tasks or testing protocols used in the laboratory are too ‘simple’ for the athlete and thus it is possible to counteract the effect of fatigue as the athlete can solely focus on task execution, with no other environmental distractions (i.e., suboptimal validity of testing). Based on our analysis of the findings related to the primary objective of this review, we have outlined our suggestions for an optimization of measuring the role of fatigue in ACL injury prevention in Sect. 4.5.

### Revised Approach

**Fig. 3:**
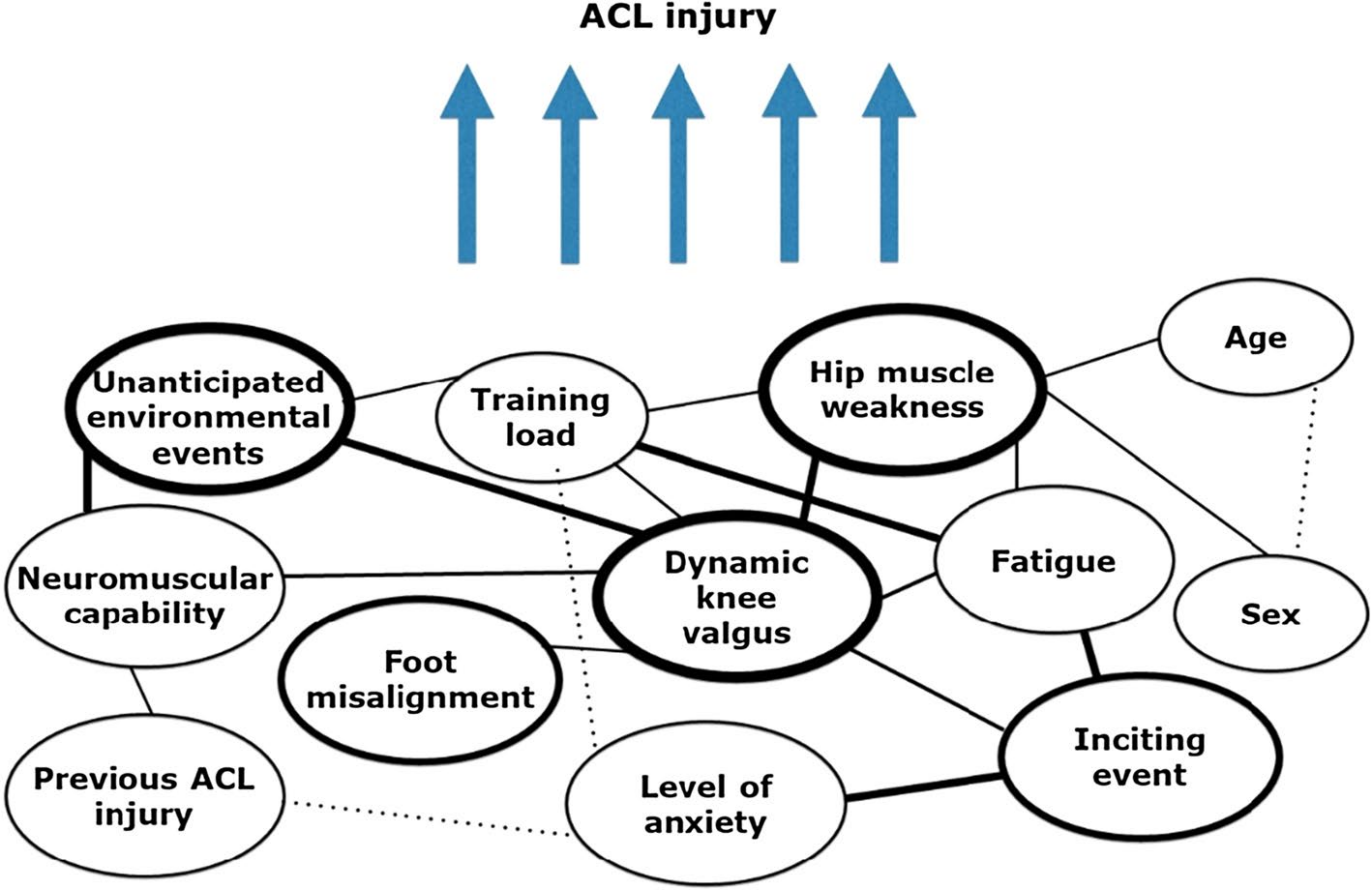
Model of complexity of factors possibly leading to ACL injury (adapted from Bittencourt et al. [80], with permission). The interaction between the various determinants is presented at the bottom of the figure. The variables that represent risk factors circled by darker lines, have more interactions and a greater influence on the outcome than variables circled by lighter lines.* ACL* anterior cruciate ligament

**Fig. 4:**
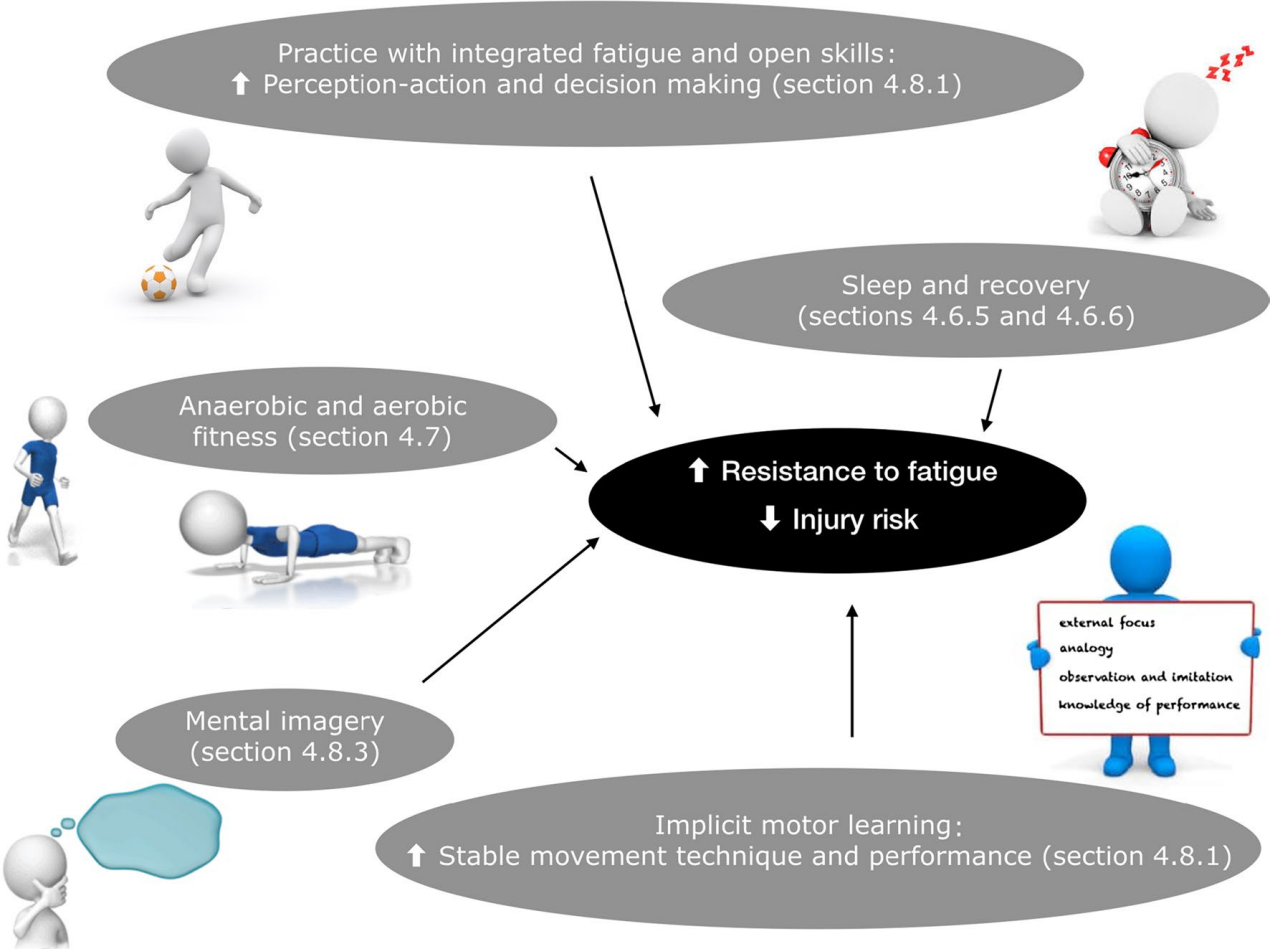
Illustration of approaches that can be considered to increase the resistance to fatigue and thus decrease injury risk

The second objective of this article was to critically appraise the current approaches in examining the effects of fatigue and propose an optimized approach of measuring the role of fatigue in ACL injury prevention to move the field forward. Even though already proposed in 2010 [27] and more recently by Bittencourt et al. [80] (‘web of determinants’, Fig. 3), inclusion of fatigue in the injury prevention paradigm has rarely been considered. Identifying isolated risk factors represents only part of the total picture and does not include the fact that an athlete’s susceptibility to injury changes dynamically [8]. There might be an underestimation of the complexity of the interaction of physical and psychological fatigue affecting neuromuscular control. When someone is fatigued, a sudden perturbation of any component of the neuromuscular system may be enough to provoke dynamic instability [8]. As an ACL injury is the result of the interaction among many different factors that can lead to vulnerability (Fig. 4), both mentally and physically [80], the complexity of the human body and brain should be appreciated.

### Proposed Approaches to Measure and Monitor Fatigue to Support Coaches

#### Protocol

General fatigue models appear to have more ecological validity in terms of simulating sports-relevant movement tasks. Applying a more general induction strategy of fatigue is therefore suggested, which may induce both peripheral and central fatigue effects [64]. It is also advised that lower extremity kinematics are quantified during the progression towards fatigue (instead of pre-post design), [15, 64] to better reflect and test the incremental effect of fatigue. Measuring the athlete’s percentage of fatigue during testing is something we would recommend as it would allow individuals to monitor the effects on landing patterns of injury prevention protocols incorporating fatigue at different intervals from pre- to post-intervention.

#### Impact of Fatigue on Decision Making

Unanticipated single-leg tasks are functionally demanding and thus high-risk movements. One leg has to adapt to the deceleration of the center of mass over a short time period, [49] which closely simulates sports-relevant movement tasks. The impact of fatigue on decision making may present a worst-case scenario for high-risk dynamic landing strategies in terms of load at the knee [45, 62, 64]. Therefore, measuring and monitoring the neuromuscular response to the impact of fatigue and decision making on injury risk should be considered within ACL injury prevention models. This also includes training of cognitive processing speed (e.g., reaction time), as this appears to be a modifiable characteristic in athletes [81].

#### Training Load

Excessive and rapid increases in training loads are likely responsible for a large proportion of non-contact soft-tissue injuries (Fig. 2) [20]. It is therefore important to monitor [internal (i.e., response to workload) and external (i.e., performed workload)] training load [82]. An increase in overall physical fitness protects the athlete against injury and serves as a moderator for decreasing the perceived workload and in turn decreasing the injury risk [17]. More specifically, there is a significant risk of injury during key stages of training and competition, such as during more intense training periods or during phases in which acute training loads change [83]. In these stages, training load and fatigue (see also Sect. 4.6.5) should be closely monitored. For example, internal load can be monitored relatively easily by measuring heart rate or by multiplying RPE by minutes practiced or played in a game (load = RPE × duration in min) [82]. It is imperative to give athletes responsibility and a voice in regulation of their perceived fatigue [8, 9].

#### Rate of Perceived Exertion

Subjective assessments through separate RPEs (e.g., Borg scale 6–20) [84] may give an indication to the peripheral load experienced, which is relevant for preventing acute injuries. An example would be to ask athletes to be specific about how much their ‘legs’ were affected, i.e., rate of perceived leg-muscle exertion (RPE-L) [23, 28]. This differentiation in physiological and biomechanical internal load enables monitoring of both central [breathlessness (RPE-B), e.g., uptake and transport of oxygen, central nervous system] and peripheral (RPE-L, e.g., neuromuscular, musculoskeletal, and muscle metabolite characteristics) exertion in team sport athletes [85].

#### Sleep

Sleep deprivation results in heightened fatigue and can elicit both psychological fatigue (perceived well-being/perceived psychological state) and physical fatigue (perceived physical state) [27, 83]. Repeated failure to obtain sufficient sleep has a cumulative detrimental effect on alertness, [27] which is necessary for attention and decision making on the field [6]. Sleep deprivation has been associated with injuries in an adolescent athletic population [86]. Fatigue, sleep quality, and feelings such as having too few breaks or not being able to obtain rest during breaks have also been identified as predictors for increased injury risk in elite soccer players [87].

#### Stress and Recovery

The importance of frequent monitoring of recovery and stress parameters to lower the risk of injuries seems to be intuitive [27, 82]. If possible, it is advised to administer the Recovery-Stress Questionnaire for Athletes (RESTQ-Sport) frequently [88]. If not possible, trainers and coaches can at least monitor stress and recovery in their athletes, for example, by asking for a simple but reliable Total Quality of Recovery Borg score (6–20) prior to a practice or game [89].

### Strategies to Delay Fatigue

Exposing the athlete to a higher chronic workload provides protection against a spike in acute workload [90, 91]. An increase in overall anaerobic and aerobic fitness may offer protection to the athlete against injury and serves as a moderator to decrease injury risk [17, 91]. This needs to be in appropriate balance with potential adverse sequelae of training (excessive fatigue, injury, illness) [17, 20]. Acute spikes in workload increase the risk of injury during a game and cause higher levels of fatigue. This fatigue can then potentially serve as a mediator, subsequently causing injury [27]. Fatigue should thus be considered as part of an injury risk profile where internal workload, aerobic fitness, and fatigue serve as interacting factors. Future research on the 3D kinematic and kinetic effects of training resistance to fatigue is warranted.

### Targeting Resistance to Fatigue

Anterior cruciate ligament injuries during ball team sports typically occur in single-leg activities such as landing on one leg or changing direction, requiring a complex coordination of peripheral and central responses [92–95]. For injury prevention, it is difficult to delineate peripheral and central fatigue mechanisms as dynamic sports maneuvers require explicit force production and motor control at both the peripheral and central (spinal and supraspinal) levels [64, 96]. However, central fatigue seems to be a critical component and targeted training of central control processes may successfully counter the impact of fatigue [45].

#### Fatigue and Decision Making

It is important to recognize the integration of fatigue and decision making as two sports-relevant factors into injury prevention programs, as this will add to the external validity and transfer of learned movement tasks to a game. Given their lack of significant impact on kinematics and kinetics, the four single-leg tasks assessed in this review may not have been sufficiently demanding (e.g., only three studies use an unanticipated design) to prevent the athlete from having enough reserve to deal with the fatigued states.

Fatigue and decision-making effects rarely exist independently of one another [64]. In addition, both central and peripheral processing mechanisms are compromised in the presence of fatigue [30, 97]. Poor perception, decision making, reactions, and resultant movement strategies may be more likely to occur when in a fatigued state. It is thus advised to include complex, sport-specific, and cognitively demanding movement tasks (i.e., open skills) in injury prevention programs as this may facilitate improved perception-action and decision making within the changing and complex sport environment [45, 64]. This can be established by including temporal constraints (e.g., time pressure for completion of a task, i.e., in dyad format, adding a competition element where one has to be faster than the other athlete), distracting the visual system (e.g., during sidestep cutting, a ball is passed to the athlete, which the player has to pass back during execution of the task), increasing the level of task uncertainty [e.g., during a vertical jump, when the athlete is in the air, he or she is given one of three options (from peer athlete or trainer, sports physical therapist) to execute immediately when landing, sprinting 45° to the left, straight ahead, or to the right], performing dual tasks and decision making (e.g., touching cones with side shuffles, where one athlete is the leader, and the other athlete follows as quickly as possible), or combinations of those factors [7].

This combination of practicing open skills in a fatigued condition where athletes have to respond to the environment will train the athlete’s ability to deal with real-world factors and stay below the injury threshold by using effective movement techniques even in a fatigued state. It is important to note that effective movement technique in a time-constrained environment with complex decision making has been shown to enhance efficient motor control with an implicit motor learning strategy [98].

#### Implicit Motor Learning: Attentional Focus

Movement technique and performance are more stable (i.e., less decline of capacity for controlling body movements) under psychological and physical stress/fatigue when acquired with an implicit learning method (e.g., external focus of attention) [99–101]. For example, research has shown that adoption of a verbal or visual external focus of attention improves biomechanics by, for example, increased knee and trunk flexion angles during cutting and landing tasks [60, 102]. In addition, neuromuscular efficiency is enhanced with implicit motor learning strategies [103–105], without a reduction in performance (e.g., jump height, force production, or shot accuracy). This is promising, as neuromuscular efficiency is particularly necessary when fatigued.

One explanation for this decreased capacity of controlling movements in a fatigued state when such movements are learned explicitly could be that integrated fatigue and decision-making effects provoke adverse movement behavior via cognitive deterioration. This progressive increase in central control increases cognitive demands [70]. Conversely, with implicit motor learning, there is no or little explicit knowledge about execution of movement, which stimulates automatic learning processes where less cognitive load is required [101, 106]. This means that when a skill is learned with an external focus of attention, more resources are available to pay attention to environmental factors [101, 106]. Thus, implicit learning may protect the athlete against the often debilitating influence of psychological or physiological stress on motor output [101].

#### Mental Imagery

Mental imagery can be an effective means to develop the central motor control strategies discussed in Sect. 4.8.2 that successfully transfer when fatigued [45]. The ability for individuals to view themselves performing correctly or making mistakes and responding to correction is of great value [101]. One theoretical approach is that learning is a problem-solving process; the more involved the individual is in analyzing his or her own performance, the greater the learning value [107]. The athlete will explore and select the solution that fits best with their body. During internal motor imagery, an athlete feels as if he/she is performing the action from a first-person visual and kinesthetic view. This replication of target movements and environmental conditions may create a “realistic” feeling as whole-body awareness is stimulated (embodied cognition) [106]. Internal imagery training may be used to implicitly improve a component of a complex motor skill [108].

In summary, mental training is associated with benefits such as decreasing stress and anxiety, increasing self-confidence, relieving pain, and increasing muscle tolerance [109]. Motor imagery techniques might thus very well be powerful in relation to experienced and/or resistance to fatigue. This can be explained by the existence of a top–down mechanism based on the activation of a central representation of the movements (instead of a peripheral focus), where spatiotemporal or dynamic control of the action is very important [110, 111].

### Study Limitations

This systematic review focused on changes in kinematics and kinetics after fatigue. Performance measures were not included in most of the included studies. The combination of both movement technique and performance (i.e., jump distance or jump height) is however important to the applied setting as the goal for athletes is to be able to stay below the injury threshold when fatigued, whilst also maintaining performance. Furthermore, in a laboratory situation, an athlete can execute movements characterized by low joint loads and reduced performance when fatigued whereas this is often not possible in real game situations, where a player has to perform maximally whilst fatigued.

Second, we analyzed the changes after fatigue per joint, and did not consider the overall body position or movement per se. This does not reflect the real world as changes in one joint affect the joint position elsewhere in the body (dynamic system). The ankle and trunk were not considered in the meta-analysis, when in fact these joints could have been used as an inter-limb compensation strategy. Additionally, frontal plane movement is lower overall and it is therefore more difficult to detect pre- vs. post-fatigue differences in this context.

The tests used were heterogeneous and different fatigue protocols (peripheral vs. central) were also used across studies. In addition, different definitions and recording of ‘peak’ angles also made it difficult to conduct a meta-analysis. For some of the outcomes, there was a small number of studies present, indicating results should be interpreted cautiously. Caution is therefore warranted when interpreting the results of this meta-analysis given the differences in definitions of fatigue, the methods used to induce fatigue, and the methods used to capture kinematics/kinetics.

The average age of included subjects was 24.89 ± 4.26 years and 20.68 ± 1.35 years for male and female subjects, respectively. This may be somewhat old for direct comparison with the population of subjects at risk [112]. The level of included athletes was mostly either recreational (i.e., practice at least three times a week for at least 30 min/day) or Division I National Collegiate Athletic Association athletes, which is comparable to the population at risk [2]. It should be noted that the type of sport was not specified in all studies. Athletes playing sports other than ball-team sports potentially have other skill levels in terms of jumping and landing and changing directions consistent with the requirements of these impact sports.

Instructions given were mostly on general task execution; only two studies indicated specifically providing verbal technical instructions (toe-to-heel strategy) [42] or not providing specific verbal technical instructions [39]. Section 4.7 highlights why instructions matter in relation to (resistance to) fatigue.

Finally, even though training under fatigued conditions has advantages and will increase the validity of the training environment in relation to the complexity of the real world, there is no consistent evidence that fatigue actually causes ACL injuries. We need to be careful about assigning a one-to-one causality.

## Conclusion

Sagittal plane variables at IC were mostly affected under the single-leg hop for distance and sidestep cutting conditions whilst peak angles were affected during a single-leg drop jump. However, fatigue had no significant impact on most of the kinetic and kinematic variables that were examined in this analysis. Given the small number of variables affected by fatigue, the question arises as to whether the fatigue pathways in play on the sports field are affected by the fatigue protocols employed in the laboratory studies included in this review. A revised approach to increase the resistance to fatigue and decrease injury risk has been proposed. For those professionals dealing with injury prevention, it is suggested to appreciate the complexity of the human body and brain and the interactions between those factors. A 50% level of fatigue in a complex environment can result in increased vulnerability to injury.

## Electronic supplementary material

Below is the link to the electronic supplementary material.

Supplementary material 1 (PDF 1259 kb)

Supplementary material 2 (PDF 755 kb)

Supplementary material 3 (PDF 1306 kb)

Supplementary material 4 (PDF 173 kb)

Supplementary material 5 (PDF 182 kb)

Supplementary material 6 (PDF 200 kb)

Supplementary material 7 (PDF 153 kb)

Supplementary material 8 (XLSX 10 kb)

Supplementary material 9 (XLSX 15 kb)

Supplementary material 10 (XLSX 9 kb)

Supplementary material 11 (XLSX 11 kb)

Supplementary material 12 (XLSX 9 kb)
