# Supplementary material for: Revised Approach to the Role of Fatigue in Anterior Cruciate Ligament Injury Prevention: A Systematic Review with Meta-Analyses
Source: Sports Med. 2019 Jan 18;49(4):565–86. doi: 10.1007/s40279-019-01052-6 (PMC6422960; doi:10.1007/s40279-019-01052-6)
Supplement: Supplementary file 3 — Supplementary material 3 (PDF 1306 kb) [file 40279_2019_1052_MOESM3_ESM.pdf]

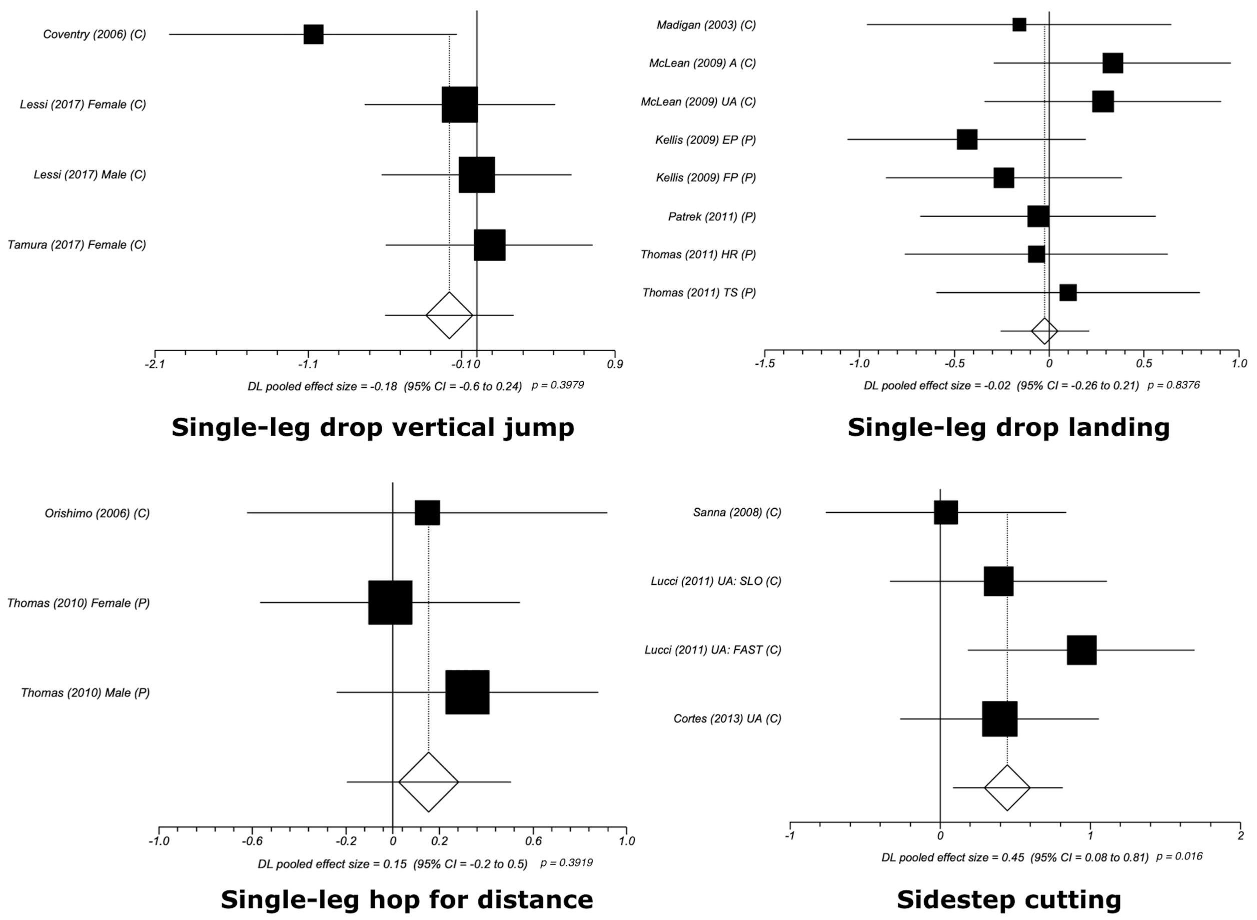

Fig. S3 Pooled effects of fatigue on hip flexion angle at initial contact (left = increased post fatigue; right = decreased post fatigue; **C = central** fatigue protocol; **P = peripheral** fatigue protocol; EP = extension protocol; FP = flexion protocol; HR = hip rotators fatigue protocol; TS = triceps surae fatigue protocol; FAST = functional agility short-term fatigue protocol; SLO = slow linear oxidative fatigue protocol; UA = unanticipated)
