# Supplementary material for: Revised Approach to the Role of Fatigue in Anterior Cruciate Ligament Injury Prevention: A Systematic Review with Meta-Analyses
Source: Sports Med. 2019 Jan 18;49(4):565–86. doi: 10.1007/s40279-019-01052-6 (PMC6422960; doi:10.1007/s40279-019-01052-6)
Supplement: Supplementary file 5 — Supplementary material 5 (PDF 182 kb) [file 40279_2019_1052_MOESM5_ESM.pdf]

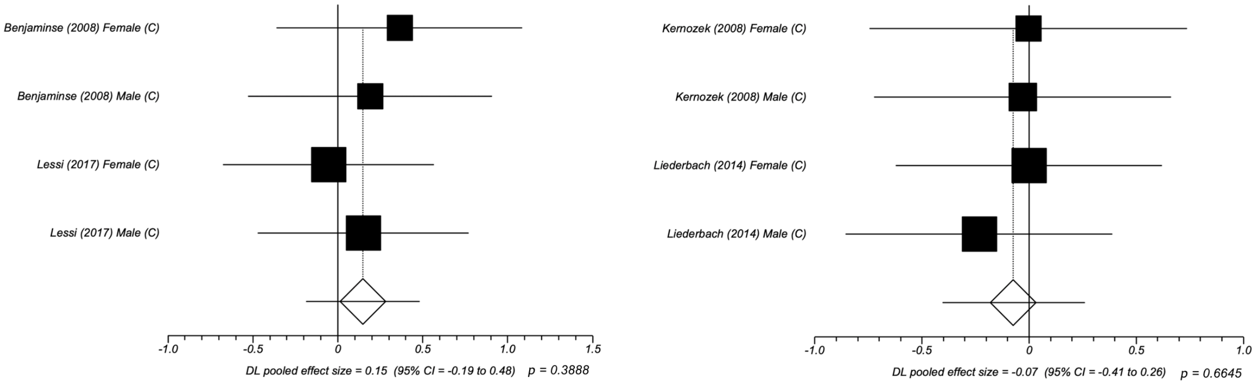

Single-leg drop vertical jump

Single-leg drop landing

Fig. S5 Pooled effects of fatigue on peak knee abduction angle (left = increased post fatigue; right = decreased post fatigue; C = central fatigue protocol; P = peripheral fatigue protocol)
